# Impact of a multifaceted antibiotic stewardship programme in a paediatric acute care unit over 8 years

**DOI:** 10.1093/jacamr/dlae181

**Published:** 2024-11-06

**Authors:** Giulia Brigadoi, Emelyne Gres, Elisa Barbieri, Cecilia Liberati, Sara Rossin, Lorenzo Chiusaroli, Giulia Camilla Demarin, Francesca Tesser, Linda Maestri, Francesca Tirelli, Elena Carrara, Evelina Tacconelli, Silvia Bressan, Carlo Giaquinto, Liviana Da Dalt, Daniele Donà

**Affiliations:** Division of Pediatric Infectious Diseases, Department for Women’s and Children’s Health, University of Padua, Via Giustiniani 3, 35128 Padua, Italy; CERPOP, UMR 1295, Inserm, University of Toulouse 3, Toulouse, France; Division of Pediatric Infectious Diseases, Department for Women’s and Children’s Health, University of Padua, Via Giustiniani 3, 35128 Padua, Italy; Division of Pediatric Infectious Diseases, Department for Women’s and Children’s Health, University of Padua, Via Giustiniani 3, 35128 Padua, Italy; Pediatric Emergency Department, Department for Women’s and Children’s Health, University of Padua, Via Giustiniani 3, 35128 Padua, Italy; Division of Pediatric Infectious Diseases, Department for Women’s and Children’s Health, University of Padua, Via Giustiniani 3, 35128 Padua, Italy; Department for Women’s and Children’s Health, University of Padua, Via Giustiniani 3, 35128 Padua, Italy; Department for Women’s and Children’s Health, University of Padua, Via Giustiniani 3, 35128 Padua, Italy; Department for Women’s and Children’s Health, University of Padua, Via Giustiniani 3, 35128 Padua, Italy; Rheumatology Unit, Department of Woman’s and Child’s Health, University of Padova, Via Giustiani 3, 35128 Padua, Italy; Division of Infectious Diseases, Department of Diagnostic and Public Health, University of Verona, Verona 37134, Italy; Division of Infectious Diseases, Department of Diagnostic and Public Health, University of Verona, Verona 37134, Italy; Pediatric Emergency Department, Department for Women’s and Children’s Health, University of Padua, Via Giustiniani 3, 35128 Padua, Italy; Division of Pediatric Infectious Diseases, Department for Women’s and Children’s Health, University of Padua, Via Giustiniani 3, 35128 Padua, Italy; Pediatric Emergency Department, Department for Women’s and Children’s Health, University of Padua, Via Giustiniani 3, 35128 Padua, Italy; Division of Pediatric Infectious Diseases, Department for Women’s and Children’s Health, University of Padua, Via Giustiniani 3, 35128 Padua, Italy

## Abstract

**Background:**

Antibiotics are the most prescribed drugs for children worldwide, but overuse and misuse have led to an increase in antibiotic resistance. Antimicrobial stewardship programmes (ASPs) have proven feasible in reducing inappropriate antimicrobial use. The study aimed at evaluating the impact and sustainability of an ASP with multiple interventions over 8 years.

**Methods:**

This quasi-experimental study was conducted between 2014 and 2022 in the paediatric acute care unit of Padua University Hospital. Demographic and clinical data were retrieved from the electronic clinical records. Daily prescriptions were collected and analysed based on the AWaRe classification and using days of therapy (DOT) out of 1000 patient days (DOT/1000PDs). The primary outcome was to assess the change in overall antibiotic consumption and of access and watch antibiotics, stratifying patients with and without comorbidities. Trends in antibiotic consumption (DOTs/1000PD) were assessed using joinpoint regression analysis.

**Findings:**

A total of 3118 children were included. Total antibiotic consumption remained stable and low in patients without comorbidities, ∼300 DOT/1000PDs, whereas a statistically significant constant reduction was observed in children with comorbidities, from almost 500 DOT/1000PPDs to <400 DOT/1000PDs. Access consumption increased in both groups of patients, whereas watch consumption constantly decreased, although statistically significant only in children with comorbidities.

**Interpretation:**

Implementing a multistep ASP has proven feasible and sustainable in improving antibiotic prescriptions for previously healthy and fragile children. All the implemented interventions were low cost, and with efficient use of resources, ensuring an ASP that was effective, practical, and easily replicable and implementable in various healthcare settings.

## Introduction

Antibiotics are among the most commonly prescribed drugs in children worldwide, both in hospital and in community settings.^1–3^ However, it has been demonstrated that up to 30% of antibiotic prescriptions are unnecessary or inappropriate, and many children receive broad-spectrum antibiotics for viral infections.^4^ While resistance can occur naturally, the excessive use of antibiotics significantly accelerates the selection of multidrug-resistant organisms.^5^ In the EU/EEU region, it is estimated that about 33 000 deaths annually can be linked to infections caused by multidrug-resistant microorganisms.^6^

In 2007, the Infectious Diseases Society of America introduced the concept of Antimicrobial stewardship programmes (ASPs) in response to the growing concern about antibiotic resistance and the limited availability of new antibiotics.^7^ ASPs aim to optimize antimicrobial use, improving clinical outcomes while minimizing toxicity, adverse events, and antimicrobial resistance. They have proven effective in reducing inappropriate antimicrobial use, enhancing patient safety, and lowering healthcare costs.^8–11^ However, in 2020, the COVID-19 pandemic disrupted antibiotic stewardship programmes globally,^12^ reducing antimicrobial stewardship activities and increasing antibiotic prescriptions due to difficulties distinguishing between viral and bacterial infections, concerns about bacterial co-infections, and the lack of effective COVID-19 treatments.

Since October 2015, a multifaceted antibiotic stewardship programme has been implemented in our department. The primary objective of the ASP was to limit the use of broad-spectrum antibiotics, such as third-generation cephalosporins, macrolides and carbapenems, which had been overprescribed indiscriminately in the years preceding its implementation. A secondary aim was to curtail unnecessary antibiotic prescriptions, thereby reducing overall antibiotic consumption.

Our study aims to assess this programme’s impact and sustainability on antibiotic prescriptions in a paediatric acute care unit (PACU) over 8 years in patients with and without comorbidities. To make results generalizable and comparable with data from other studies, we used the AWaRe classifications and metrics developed in 2017 by the WHO Expert Committee on Selection and Use of Essential Medicines, updated in 2023.^8^ The AWaRe classification is a simple traffic-light system to improve the utilization of lower class (access) antibiotics, making the basis for ASPs.^13^

## Materials and methods

### Setting, study design and population

This quasi-experimental study was conducted at the PACU for Women’s and Children’s Health at Padua University Hospital from 1 October 2014 to 31 December 2022. The hospital provides primary and secondary care to a metropolitan area of 350 000 people, of which 45 000 are younger than 15 years, and tertiary care to the regional and extra-regional population. The paediatric emergency department accounts for ∼25 000 visits per year, with a 7% rate of admission to hospital. During the COVID-19 pandemic, PACU was reorganized to host children and parents with SARS-CoV-2 infections.

**Figure 1.**
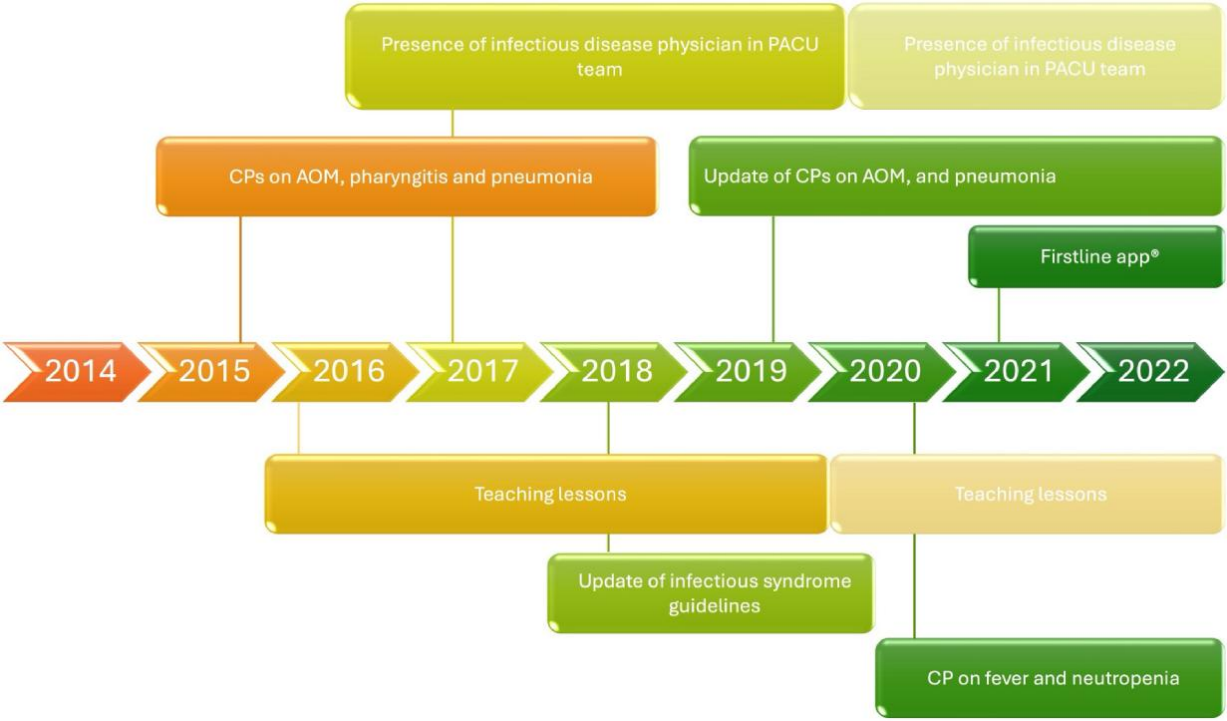
The sequential intervention of the multifaceted ASP through the 8 years. More transparent colours represent the COVID-19 period when many educational activities were either reduced or conducted remotely, and the presence of infectious disease physician at ward rounds was reduced due to pandemic-related restrictions. CPs, clinical pathways; AOM, acute otitis media; PACU, paediatric acute care unit.

The 8 years of the study were divided into 3 periods:

- Pre-implementation period, from October 2014 to September 2015, before the start of the implementation of the ASP
- Post-implementation period, from October 2015 to March 2020, after the implementation of the sequential multifaceted ASP [clinical pathways (CPs) and internal guidelines, educational sessions, the presence of infectious disease physicians at the ward round see Figure 1 and Supplementary material (available as Supplementary data at *JAC-AMR* Online) for the details of the programme]
- COVID-19 pandemic period, from April 2020 to December 2022, during which a mobile app with all the internal guidelines was developed and released to all residents and consultants

The cohort study consisted of all patients admitted to the PACU. For children receiving antibiotics, we extracted additional data on clinical characteristics and comorbidities. Children already admitted to other hospitals or other wards and transferred to the PACU with ongoing antibiotic therapy were excluded from the analysis. Patients transferred to different wards or hospitals were lost to follow-up after the day of transfer.

We followed the Strengthening the Reporting of Observational Studies in Epidemiology reporting guidelines.^14^

### Definition of exposure

Therapeutic antibiotic prescriptions were classified according to the 2023 WHO AWaRe classification (Access, Watch and Reserve classification) and the antibiotic classes (penicillin, cephalosporin, aminoglycosides, etc.).

### Data

Patients receiving antibiotics were identified by consulting electronic records. For these patients, pseudoanonymized clinical, demographic, diagnostic and daily prescription data were manually collected from electronic medical records using a password-protected REDCap data collection form. Regarding prescription data, type of antibiotic, route of administration, dosage, start and end of therapy, and reason for ending or changing treatment were collected for each patient. The study was approved by the Institutional Review Board of Padua University Hospital (protocol number 295n/AO/22).

### Outcomes

The primary outcome of the study was to assess the change in overall antibiotic consumption and consumption of access and watch antibiotics over 8 years, stratifying patients without comorbidities and those with at least one comorbidity.

The secondary outcomes were to assess the change in clinical outcomes in the three different periods considering length of stay (LOS) in hospital, number of paediatric intensive care unit (PICU) admissions and number of deaths, the number of prescriptions divided by antibiotic categories in the three study periods and the change in total antibiotic use according to the AWaRe classes in children admitted for lower respiratory tract infections (LRTIs), including bronchiolitis, community-acquired pneumonia, complicated pneumonia, interstitial pneumonia, wheezing, aspiration pneumonia and uncomplicated or complicated upper respiratory tract infections (URTI and complicated URTI), including pharyngitis, otitis, sinusitis, rhinitis in neonates, mastoiditis and deep neck abscess as complications. We considered these two groups of infectious diseases as they were the most frequent causes of admission to PACU.

### Statistical analysis

The results in the different periods were summarized as numbers and percentages (categorical variables), median, 25° and 75° interquartile. In a contingency table, categorical variables were compared with χ^2^ or Fisher’s two-tailed exact test and continuous variables with the non-parametric Kruskal–Wallis rank-sum test.

Discharge diagnoses were gathered in 12 different classes, and >1 diagnosis per child was possible. Since not all discharge diagnoses were part of these classes, another class, identified as ‘other’, was added.

Data were gathered in 33 trimesters for the analysis of all the patients, in 16 semesters (from January 2015 to December 2022) for LRTI and uncomplicated and complicated URTI.

Diagnoses and classes of antibiotics prescribed were described using bar charts.

Antibiotic consumption was evaluated by days of therapy/1000 patient days (DOT/1000PD). DOTs were computed from all patients receiving antimicrobial therapies, and patient days were calculated based on the overall ward admissions or stratifying by diagnoses.

Trends in antibiotic prescribing (DOTs/1000PD) were assessed using joinpoint regression analysis. An algorithm tests whether trends in the quarter percentage change (QPC) or biannualy percentage change (BPC) of antibiotic use fit a series of joined straight lines on a logarithmic scale. The line segments converge at specific points, called joinpoints, which indicate a change in trend.^15^ The weighted Bayesian Information Criterion method was used to identify the number of significant joinpoints. Using the calendar quarter or semester as a variable, the joinpoint regression analysis estimated the QPC or BPC of the rates between each joinpoint and the average QPC or BPC with a 95% CI.^16^ We estimated the autocorrelation of our data using an autocorrelogram included in the model.

The analyses were conducted using R.^17^ The jointpoint regression analyses were performed using the Joinpoint software version 5.0.2 (May 2023; Division of Cancer Control and Population Sciences, National Cancer Institute).^18^  *P* < 0.05 was considered statistically significant.

## Results

During the study period, 7229 patients were admitted to PACU, of whom 3118 received antibiotic therapy (43.1%): 387 in the pre-implementation period, 1881 in the post-implementation period and 850 in the COVID-19 pandemic period. Table 1 displays the demographic and clinical characteristics of patients receiving antibiotics. The number of patients with comorbidities remained substantially similar between the pre- and post-implementation periods (36.7% and 33.2%, respectively), whereas it increased to 42.9% in the COVID-19 pandemic period.

**Table 1.** Demographic, clinical characteristics and outcomes of included patients

	Pre-implementation	Post-implementation	COVID-19 pandemic	Total	*P*-value
Total admission	739	4293	2197	7229	
Total patients with antibiotic prescription	387 (52.4%)	1881 (43.8%)	850 (38.7%)	3118 (43.1%)	<0.00001
Median age (months and 25–75°)	25.8 (4.3, 69.6)	31.3 (7.1, 78.0)	32.6 (8.0, 92.9)	31.1 (6.9, 80.1)	0.069
Number of patients per age					0.038
≤30 days	35 (9.0%)	147 (7.8%)	84 (9.9%)	266 (8.5%)	
1–3 months	47 (12.1%)	170 (9.0%)	75 (8.8%)	292 (9.4%)	
4–12 months	57 (14.7%)	278 (14.8%)	102 (12.0%)	437 (14.0%)	
1–5 years	158 (40.8%)	779 (41.4%)	329 (38.7%)	1266 (40.6%)	
6–10 years	51 (13.2%)	284 (15.1%)	133 (15.6%)	468 (15.0%)	
11–14 years	33 (8.5%)	174 (9.3%)	90 (10.6%)	297 (9.5%)	
>15 years	6 (1.6%)	49 (2.6%)	37 (4.4%)	92 (3.0%)	
Sex					0.138
Female	151 (39.0%)	814 (43.3%)	383 (45.1%)	1348 (43.2%)	
Male	236 (61.0%)	1067 (56.7%)	467 (54.9%)	1770 (56.8%)	
Comorbidities (%)					<0.001
No comorbidities	245 (63.3%)	1256 (66.8%)	485 (57.1%)	1986 (63.7%)	
At least one comorbidity	142 (36.7%)	625 (33.2%)	365 (42.9%)	1132 (36.3%)	
LOS (months and 25–75°)					
No comorbidities	6.0 (4.0, 7.0)	5.0 (4.0, 7.0)	4.0 (3.0, 6.0)	5.0 (4.0, 7.0)	<0.001
At least one comorbidity	6.0 (4.0, 9.0)	6.0 (4.0, 8.0)	5.0 (3.0, 8.0)	5.0 (4.0, 8.0)	<0.001
PICU admission (%)					
No comorbidities	2 (0.8%)	15 (1.2%)	7 (1.4%)	24 (1.2%)	0.823
At least one comorbidity	3 (2.1%)	27 (4.3%)	16 (4.4%)	46 (4.1%)	0.484
Death (%)					
No comorbidities	0	0	0	0	
At least one comorbidity	0 (0.0%)	0 (0.0%)	1 (0.3%)	1 (0.1%)	0.452

LOS, length of stay; PICU, paediatric intensive care unit.

**Figure 2.**
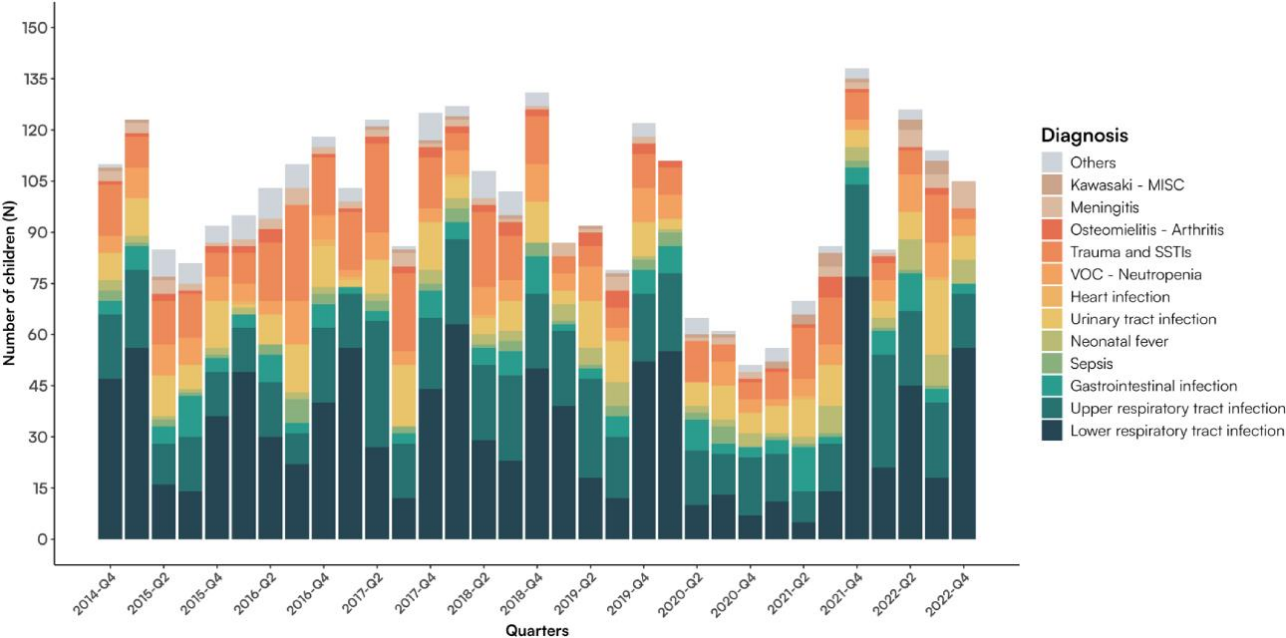
Diagnoses presented by quarter between 2014 and 2022 in children admitted to PACU at Padua Hospital. MISC, multisystem inflammatory syndrome in children; SSTIs, skin and soft tissue infections; VOC, vasoocclusive crisis.

URTI and LRTI were the most common diagnoses, especially in the trimesters between September and March. In 2020–21, the number of admissions to the PACU decreased, and URTI and LRTI remained lower than in previous years, showing no increase in the autumn-winter periods. Figure 2 presents the diagnoses in the different quarters.

### Main outcome

**Figure 3.**
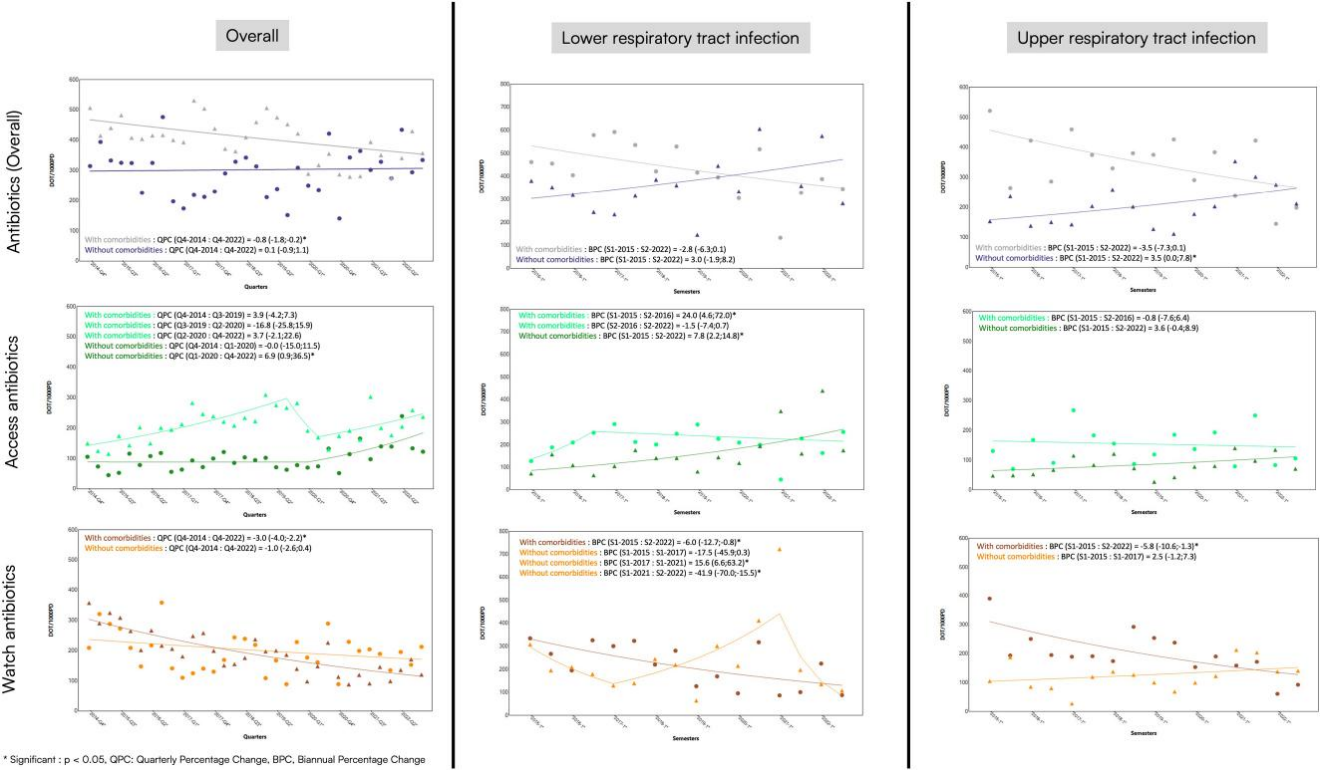
The analyses of prescription trends between 2014 and 2022 for all antibiotics by quarter and between 2015 and 2022 for URTI and LRTI by half-year (joinpoint analysis). DOT/1000PD, days of therapy/1000 patient days; Q1–4, quarters S1–4 semesters.

Overall, the DOT/1000PDs for total antibiotic consumption remained stable during the 8 years in patients without comorbidities, ∼300 DOT/1000PDs, whereas a statistically significant constant reduction was observed in patients with comorbidities, from almost 500 DOT/1000PPDs to <400 DOT/1000PDs (decrease of 0.8 DOT/1000PDs each trimester, 95% CI −1.8, −0.2; Figures 3 and S1).

Considering the type of antibiotics prescribed, classified based on the AWaRe classification, a different pattern was observed for access and watch antibiotics. Access DOT/1000PDs increased in patients with and without comorbidities. Two significant changes in trend were detected for patients with comorbidities, which increased until the beginning of 2020, followed by a decrease during the first months of the COVID-19 pandemic and a new increase in the months after. None of these changes is statistically significant. Only one joinpoint was detected for patients without comorbidities. No difference in DOT/1000PDs was observed until 2020, whereas since 2020, a statistically significant increase in DOT/1000PDs was detected (increase of 6.9 DOT/1000PDs each trimester, 95% CI 0.9, 36.5).

Watch DOT/1000PDs constantly decreased for patients with and without comorbidities, but only the former had a statistically significant reduction (reduction of 3 DOT/1000PDs each trimester, 95% CI −4, −2.2; Figures 3 and S1).

### Secondary outcomes

#### Clinical outcomes

The median LOS in the hospital showed a statistically significant decrease both in children with and without comorbidities, from 6 to 4 days and from 6 to 5 days, respectively. No difference in PICU admission and number of deaths was observed both in children with and without comorbidities (Table 1).

#### Antibiotic categories prescriptions

**Figure 4.**
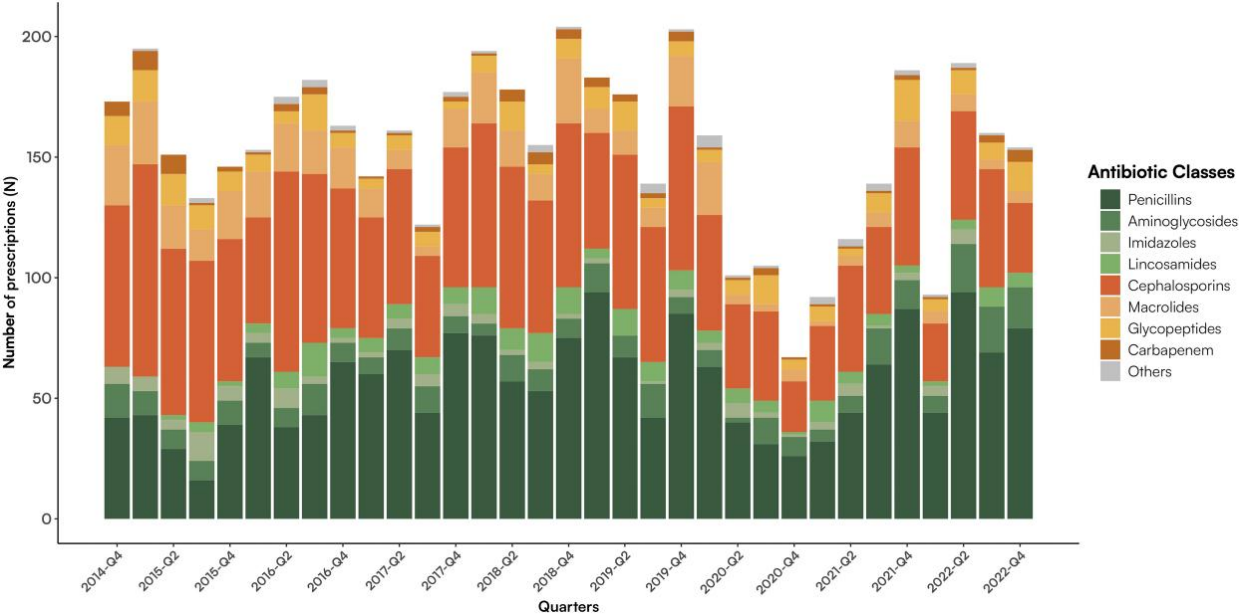
Class of antibiotics prescribed per quarter between 2014 and 2022 in children admitted to PACU at Padua Hospital.

Cephalosporins and penicillin were the most commonly prescribed antibiotics across all trimesters. The distribution of antibiotic types in each trimester is illustrated in Figure 4.

##### LRTI

In patients with LRTI, DOT/1000PDs showed a slight, non-significant increase in those without comorbidities and a decrease in those with comorbidities. Access DOT/1000PDs consistently increased significantly in patients without comorbidities (increase of 7.8 DOT/1000PDs each trimester, 95% CI 2.2, 14.8), and had a significant increase from 2015 to 2016 in patients with comorbidities (increase of 24 DOT/1000PDs each trimester, 95% CI 4.6, 72). Watch DOT/1000PDs significantly decreased in patients with comorbidities (reduction of 6 DOT/1000PDs each trimester, 95% CI −12.7, −0.8), while in patients without comorbidities, it initially decreased non-significantly, then significantly increased until 2021, followed by a significant reduction (decrease of 41.9 DOT/1000PDs, 95% CI −70.0, −15.5; Figures 3 and S2).

##### Uncomplicated and complicated URTI

For patients with uncomplicated and complicated URTI, total DOT/1000PDs significantly increased in those without comorbidities and slightly decreased in those with comorbidities, though not significantly. Access DOT/1000PDs remained stable in both groups. Watch DOT/1000PDs significantly decreased in patients with comorbidities, while a slight, non-significant increase was observed in patients without comorbidities (Figures 3 and S3).

## Discussion

Our study showed the efficacy and sustainability of a multifaceted antibiotic stewardship programme in a PACU, considering patients with and without comorbidities. CPs, face-to-face lessons, the presence of the PID at the ward round, and the mobile app Firstline^®^ were quickly integrated into the daily clinical practice of residents and senior doctors.

The three phases identified in the study served as a comparison between the stewardship period, from October 2015 to March 2020, with a previous period in which no ASP was active (October 2014–September 2015) and with a subsequent period during which the pandemic disrupted some ongoing activities (April 2020–December 2022).^19^

DOT/1000PDs for both total antibiotic prescriptions and watch antibiotics showed a greater decrease in children with comorbidities compared with those without. However, antibiotic use in children without comorbidities was already low compared with other studies.^19^ Despite guidelines mainly targeting healthy children, physicians may have become more cautious about antibiotic use in children with comorbidities, who often received antibiotics for viral infections due to their underlying conditions. Educational initiatives and the presence of PID physicians likely contributed to reducing inappropriate antibiotic prescriptions in these vulnerable children.^20–22^

Access DOT/1000PDs in fragile children increased from 2015 to the end of 2019, indicating consistent improvement post-intervention. During the initial months of the COVID-19 pandemic, both Access antibiotic prescriptions and total antibiotic use decreased, aligning with trends observed in other studies.^20,21^ Despite lower SARS-CoV-2 infection rates in children, those with comorbidities might have received antibiotics for mild COVID-19 cases.^22^ Reduced admissions for viral infections, due to decreased respiratory virus circulation, and possibly more bacterial infection-related admissions, might contribute to the observed antibiotic prescription trends. Contrary, in children without comorbidities access DOT/1000PDs increased during the COVID-19 pandemic. While global antibiotic stewardship programmes were generally disrupted, our study showed a different outcome. Although there was an initial halt in educational activities and PID participation in the ward round, these interventions were resumed online and supported by the Firstline^®^ app, which made internal guidelines more accessible to residents and consultants.

Despite the decrease in overall antibiotic use and watch antibiotics, and the increase in access antibiotics, no increase in deaths and number of patients admitted to PICU was observed compared with the pre-intervention group, underscoring the effectiveness of the ASP interventions. Instead, a reduction in the length of hospital stays post-implementation and during the pandemic was noted, particularly in children without comorbidities, highlighting the success of the interventions in optimizing antibiotic therapy duration in the hospital.

The change in antibiotic use for patients admitted with LRTI was more pronounced in those with comorbidities, showing a significant reduction in watch DOT/1000PDs, compared with healthy patients, who exhibited a fluctuating trend. Fragile patients were often treated with antibiotics like ceftriaxone and clindamycin for aspiration pneumonia.^23^ Over the 8 years, the increasing use of amoxicillin-clavulanate contributed to the reduction in watch antibiotic consumption.

Considering uncomplicated and complicated URTIs, the change in antibiotic use was more evident in children with comorbidities compared with healthy children. Conservative management instead of surgical intervention for conditions like mastoiditis and deep neck abscesses increased throughout the 8 years, leading to more antibiotic therapy cases in the post-implementation periods.^24,25^ Conversely, it is possible that vulnerable children were admitted for monitoring even with mild viral infections, potentially explaining the statistical reduction in antibiotic use, particularly in watch DOT/1000PDs.

To our knowledge, this is the first study that evaluated the impact of a multifaceted stewardship programme implemented in a general paediatric acute care ward without excluding patients with comorbidities. Children with chronic conditions are usually not considered in this type of programme unless the intervention is tailored to them, such as the implementation of guidelines regarding fever in neutropenia. Fragile patients are more frequently treated with antibiotics, even in case of suspected viral infection, due to the increased fear of possible infectious disease complications. We did not include specific guidelines for children with chronic conditions, but it is possible that physicians were sensitive to prescribe fewer antibiotics in case of suspected viral infections, considering that children could be monitored in the ward.

Additionally, these interventions were low cost, ensuring that the desired health outcomes were achieved efficiently by minimizing unnecessary expenditures and optimizing resource utilization. Consequently, these interventions may be not only effective but also practical, making them easily replicable and implementable across diverse healthcare settings. This adaptability is crucial for broader public health initiatives, as it ensures that other institutions, regardless the resource availability, can adopt these strategies to achieve similar improvements in antibiotic prescribing practices and patient outcomes.

Our study has several limitations. First, it was a retrospective study, with a manual collection of daily data. Other changes, such as infection prevention and control measures, changes in national guidelines, and the availability of antibiotics, could have influenced the changes in our antibiotic use. Second, we did not evaluate the impact of this multifaceted ASP on antibiotic adverse events or clinical outcomes in the 30 days after discharge. Finally, our antibiotic stewardship programme consisted of multiple types of intervention implemented at different times over the 8 years, altogether pointing to the same purpose: providing clear and updated information regarding the most common infectious diseases, both treated as outpatients and inpatients, and sensibilize paediatricians to appropriate antibiotic use. For this reason, it was not possible to assess the efficacy of each single intervention. However, different studies have already shown the efficacy of these interventions.^11,26–32^

### Conclusions

A multistep ASP has proven both feasible and sustainable in improving antibiotic prescriptions among healthy and fragile paediatric patients. Our study demonstrates that physicians’ awareness and sensitization to responsible antimicrobial prescribing extend benefits to other populations not initially targeted by the intervention, such as children with comorbidities. Key components include continuing education through educational sessions and frequent reminders, easily accessible electronic guidelines platforms, and active discussions with paediatric infectious disease specialists during ward rounds. All the implemented interventions were low cost and used resources efficiently, ensuring an ASP that was not only effective but also practical. These interventions were easily replicable and implementable in various healthcare settings, thereby supporting broader public health initiatives to improve antibiotic prescribing practices and patient outcomes. Future prospects include the standardization of stewardship strategies with the incorporation of network centres and outpatient health providers.

## Supplementary Material

dlae181_Supplementary_Data

## Data Availability

The data used in this study cannot be made publicly available due to Italian data protection laws. The anonymized data sets generated during the current study can be provided on request, from the corresponding author, after written approval by the local ethic committee.

## Supplementary data

Figure S1 is available as Supplementary data at *JAC-AMR* Online.

## References

[1] Jackson C, Hsia Y, Bielicki JA et al Estimating global trends in total and childhood antibiotic consumption, 2011–2015. BMJ Glob Health 2019; 4: e001241. 10.1136/bmjgh-2018-001241 PMC6407570 30899565

[2] Browne AJ, Chipeta MG, Haines-Woodhouse G et al Global antibiotic consumption and usage in humans, 2000–18: a spatial modelling study. Lancet Planet Health 2021; 5: e893–904. 10.1016/S2542-5196(21)00280-1 34774223 PMC8654683

[3] Gerber JS, Newland JG, Coffin SE et al Variability in antibiotic use at children’s hospitals. Pediatrics 2010; 126: 1067–73. 10.1542/peds.2010-1275 21078728 PMC4677056

[4] Zetts RM, Stoesz A, Smith BA et al Outpatient antibiotic use and the need for increased antibiotic stewardship efforts. Pediatrics 2018; 141: e20174124. 29793986 10.1542/peds.2017-4124

[5] Levy SB . Factors impacting on the problem of antibiotic resistance. J Antimicrob Chemother 2002; 49: 25–30. 10.1093/jac/49.1.25 11751763

[6] European Centre for Disease Prevention and Control . *Assessing the Health Burden of Infections with Antibiotic-Resistant Bacteria in the EU/EEA, 2016–2020*. 2020. https://www.ecdc.europa.eu/en/publications-data/health-burden-infections-antibiotic-resistant-bacteria-2016-2020.

[7] Dellit T, Owens R, McGowan JJ et al Infectious Diseases Society of America and Society for Healthcare Epidemiology of America guidelines for developing an institutional program to enhance antimicrobial stewardship. Clin Infect Dis 2007; 44: 159–77. 10.1086/510393 17173212

[8] Rossin S, Barbieri E, Cantarutti A et al Multistep antimicrobial stewardship intervention on antibiotic prescriptions and treatment duration in children with pneumonia. PLoS One 2021; 16: e0257993. 10.1371/journal.pone.0257993 34705849 PMC8550372

[9] Barbieri E, Donà D, Cantarutti A et al Antibiotic prescriptions in acute otitis media and pharyngitis in Italian pediatric outpatients. Ital J Pediatr 2019; 45: 103. 10.1186/s13052-019-0696-9 31420054 PMC6697973

[10] Donà D, Barbieri E, Daverio M et al Implementation and impact of pediatric antimicrobial stewardship programs: a systematic scoping review. Antimicrob Resist Infect Control 2020; 9: 1–12. 10.1186/s13756-019-0662-8 31911831 PMC6942341

[11] Donà D, Zingarella S, Gastaldi A et al Effects of clinical pathway implementation on antibiotic prescriptions for pediatric community-acquired pneumonia. PLoS One 2018; 13: e0193581. 10.1371/journal.pone.0193581 29489898 PMC5831636

[12] Ashiru-Oredope D, Kerr F, Hughes S et al Assessing the impact of covid-19 on antimicrobial stewardship activities/programs in the United Kingdom. Antibiotics 2021; 10: 1–13. 10.3390/antibiotics10020110 PMC7912640 33498716

[13] WHO . AWaRe Classification of Antibiotics for Evaluation and Monitoring of Use. https://www.who.int/publications/i/item/WHO-MHP-HPS-EML-2023.04.

[14] von Elm E, Altman DG, Egger M et al The Strengthening the Reporting of Observational Studies in Epidemiology (STROBE) statement: guidelines for reporting observational studies. J Clin Epidemiol 2008; 61: 344–9. 10.1016/j.jclinepi.2007.11.008 18313558

[15] Kim HJ, Fay MP, Feuer EJ et al Permutation tests for joinpoint regression with applications to cancer rates. Stat Med 2000; 19: 335–51. 10.1002/(sici)1097-0258(20000215)19:3<335::aid-sim336>3.0.co;2-z 10649300

[16] Clegg LX, Hankey BF, Tiwari R et al Estimating average annual per cent change in trend analysis. Stat Med 2009; 28: 3670–82. 10.1002/sim.3733 19856324 PMC2843083

[17] R Core Team, R Foundation for Statistical Computing, Vienna, Austria . *R: A Language and Environment for Statistical Computing*. 2023. https://www.R-project.org/.

[18] Statistical Methodology and Applications Branch, Surveillance Research Program, National Cancer Institute . Joinpoint Regression Program, Version 5.2.0.0. 2024.

[19] Zaffagnini A, Rigotti E, Opri F et al Enforcing surveillance of antimicrobial resistance and antibiotic use to drive stewardship: experience in a paediatric setting. J Hosp Infect 2024; 144: 14–9. 10.1016/j.jhin.2023.12.001 38092304

[20] Castro-Lopes A, Correia S, Leal C et al Increase of antimicrobial consumption in a tertiary care hospital during the first phase of the covid-19 pandemic. Antibiotics 2021; 10: 778. 10.3390/antibiotics10070778 34202340 PMC8300755

[21] Park JY, Kang HM. National level cross-sectional study on antibiotic use in children during the pre- and early COVID-19 eras. Antibiotics 2024; 13: 249. 10.3390/antibiotics13030249 38534684 PMC10967617

[22] Bassi F, Doria M. Diffusion of COVID-19 among children and adolescents during the second and third waves of the pandemic in Italy. Eur J Pediatr 2022; 181: 1619–32. 10.1007/s00431-021-04330-6 35083537 PMC8791678

[23] Thomson J, Hall M, Ambroggio L et al Antibiotics for aspiration pneumonia in neurologically impaired children. J Hosp Med 2020; 15: 395–402. 10.12788/jhm.3338 31891564 PMC7641495

[24] Loh R, Phua M, Shaw CKL. Management of paediatric acute mastoiditis: systematic review. J Laryngol Otol 2018; 132: 96–104. 10.1017/S0022215117001840 28879826

[25] Wilkie MD, De S, Krishnan M. Defining the role of surgical drainage in paediatric deep neck space infections. Clin Otolaryngol 2019; 44: 366–71. 10.1111/coa.13315 30784193

[26] Dona D, Baraldi M, Brigadoi G et al The impact of clinical pathways on antibiotic prescribing for acute otitis media and pharyngitis in the emergency department. Pediatr Infect Dis J 2018; 37: 901–7. 10.1097/INF.0000000000001976 29561517

[27] Barbieri E, De Luca M, Minute M et al Impact and sustainability of antibiotic stewardship in pediatric emergency departments: why persistence is the key to success. Antibiotics (Basel) 2020; 9: 867. 10.3390/antibiotics9120867 33291565 PMC7761799

[28] Rimawi RH, Mazer MA, Siraj DS et al Impact of regular collaboration between infectious diseases and critical care practitioners on antimicrobial utilization and patient outcome. Crit Care Med 2013; 41: 2099–107. 10.1097/CCM.0b013e31828e9863 23873275

[29] Saunderson RB, Gouliouris T, Cartwright EJ et al Impact of infectious diseases consultation on the management of *Staphylococcus aureus* bacteraemia in children. Open 2014; 4: e004659. 10.1136/bmjopen-2013-004659 PMC4091395 24989617

[30] Shawki MA, AlSetohy WM, Ali KA et al Antimicrobial stewardship solutions with a smart innovative tool. J Am Pharm Assoc 2021; 61: 581–8.e1. 10.1016/j.japh.2021.04.013 33962893

[31] Charani E, Gharbi M, Moore LSP et al Effect of adding a mobile health intervention to a multimodal antimicrobial stewardship programme across three teaching hospitals: an interrupted time series study. J Antimicrob Chemother 2017; 72: 1825–31. 10.1093/jac/dkx040 28333297 PMC5437525

[32] Primhak S, Pool N, Sam MSY et al Improved paediatric antimicrobial prescribing with a smartphone application: a before and after interventional study. Arch Dis Child 2023; 108: 899–903. 10.1136/archdischild-2023-325795 37463738

