# Supplementary material for: Impact of a multifaceted antibiotic stewardship programme in a paediatric acute care unit over 8 years
Source: JAC Antimicrob Resist. 2024 Nov 6;6(6):dlae181. doi: 10.1093/jacamr/dlae181 (PMC11538966; doi:10.1093/jacamr/dlae181)

**Supplementary material**

Type of interventions implemented

This is a multifaceted antimicrobial stewardship program over eight years, started in October 2015. The program involved the Pediatric Acute Care Unit (PACU) teams, considering both senior doctors and residents working every day in the ward, an ID physician and after 2020 the figure of clinical pharmacist, who provided advice in the more complex situation, considering possible interaction in case of polydrug therapy especially with antiviral treatment for COVID-19.

*1. Clinical Pathways*

The first intervention started in October 2015, with the introduction of Clinical Pathways (CP) for managing acute otitis media (AOM), pharyngitis, and community-acquired pneumonia (CAP). These first CPs were developed in collaboration with the Children’s Hospital of Philadelphia. CP is a one-page decision algorithm that guides physicians through the most important steps in caring for patients with a specific infectious syndrome. It summarized international guidelines for the diagnosis and treatment of the specific condition. Two CPs (AOM and CAP) were updated in December 2019, based on the new evidence. Regarding this last update for CAP, two new steps were introduced: one regards the treatment of bronchiolitis, with the recommendation to avoid antibiotic treatment unless a concomitant bacterial infection is suspected, and the second regards the use of procalcitonin (PCT) both to decide if antibiotics are needed and to evaluate the length of therapy in hospital based on the results of PCT after 72 hours comparing it to the values of PCT at admission. More information regarding CP has been already published^1–4^.

In December 2020, a new CP regarding the management of fever in neutropenia was introduced. This CP guided the physician in choosing the better antibiotic therapy based on the neutrophilic count of each patient. Furthermore, piperacillin-tazobactam, instead of ceftazidime, was recommended as first-line treatment in combination with amikacin and eventually a glycopeptide. This change was made due to the high potential of cephalosporins to lead to the development of MDR bacteria.

CPs were presented to PED and PACU residents and senior doctors. Training sessions and educational lectures were organized yearly until March 2020 to explain the guidelines and the rationale of CP to all physicians and residents. CP pocket cards were delivered to all physicians, and CP posters were hung on PED and PACU walls in 2015 and with the update in 2019.

*2. Internal Guidelines*

Internal Guidelines (IGs) are written by experts and updated every three to four years. They provide physicians with information about epidemiology, clinical manifestations, diagnostic tools, and therapeutic options. Since 2017, the IGs for the most common pediatric infectious diseases have been updated and made available to all residents and senior doctors on the hospital intranet.

*3. Teaching lessons*

Padua Hospital is a University Hospital, and face-to-face lessons are an essential part of the residency programs. Educational talks regarding different pediatric topics are organized twice per week, between October and June, and are held by experts in the field and residents. Since 2016, lessons regarding the most common pediatric infectious disease syndromes have been organized. Due to the COVID-19 pandemic, all the teaching meetings were initially suspended and restarted after a couple of months online.

*4. Introduction of the role of infectious disease physician in the multidisciplinary team of the PACU*

Between 2017 and March 2020, the role of infectious disease physician (ID physician) was added to the multidisciplinary PACU’s team and became a pivotal figure during the ward rounds. The ID physician attended at least two ward rounds every week and the multidisciplinary meetings about patients with infectious diseases. At first, the intervention aimed at reducing the use of Watch agents, such as meropenem, and choosing the optimal agents for each different infectious disease, explaining the rationale of the recommendations; secondly at changing antibiotic prescriptions and targeting them to microbiological isolation, choosing the optimal agents, and the best dosage based on antibiograms and the site of infection; third, at evaluating the correct switch from parenteral to oral therapy and the proper therapy duration, recommending these changes to the ward’s physicians when not considered.

During the COVID-19 period, the infectious disease physician could not regularly participate in ward rounds due to restriction rules but discussed the most severe cases via telemedicine.

*5. Firstline® app*

In April 2021, an app to help physicians choose the best empirical antibiotic therapy for each of the most common infectious diseases was made available to all physicians and residents in pediatric wards. Firstline® is an app built by physicians and residents of the pediatric department to maximize the spread of the best local practices. This app contains and summarizes the internal guidelines, the clinical pathways, and the teaching lessons already published and presented to all Padua residents and senior doctors, making them available with a simple touch on the phone.

*References*

1. Donà D, Zingarella S, Gastaldi A, *et al.* Effects of clinical pathway implementation on antibiotic prescriptions for pediatric community-acquired pneumonia. *PLoS One* 2018; **13**: 1–15.

2. Dona D, Baraldi M, Brigadoi G, *et al.* The Impact of Clinical Pathways on Antibiotic Prescribing for Acute Otitis Media and Pharyngitis in the Emergency Department. *Pediatr Infect Dis J* 2018; **37**: 901–7.

3. Rossin S, Barbieri E, Cantarutti A, *et al.* Multistep antimicrobial stewardship intervention on antibiotic prescriptions and treatment duration in children with pneumonia. *PLoS One* 2021; **16**: 1–14.

4. Barbieri E, De Luca M, Minute M, *et al.* Impact and sustainability of antibiotic stewardship in pediatric emergency departments: Why persistence is the key to success. *Antibiotics* 2020; **9**: 1–16.


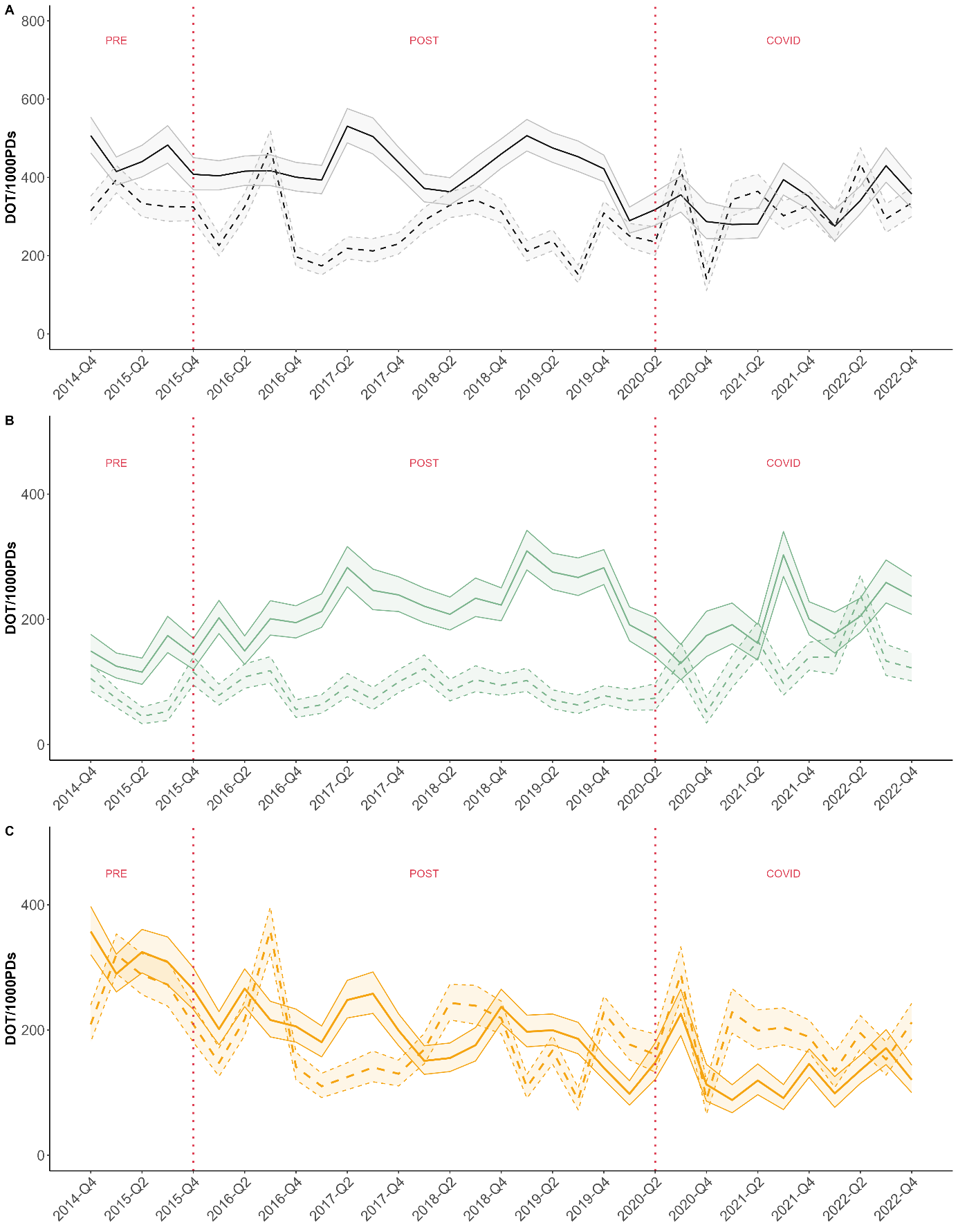
**Figure S1.** Evolution of DOT/1000 PDs for all the antibiotics (A) and according to the AWaRe classification: Access (B) and Watch (C) by quarters during the study period (2014-2022).

With comorbidities (CI95%)

Without comorbidities (CI95%)

**Figure S2.**
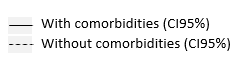
**
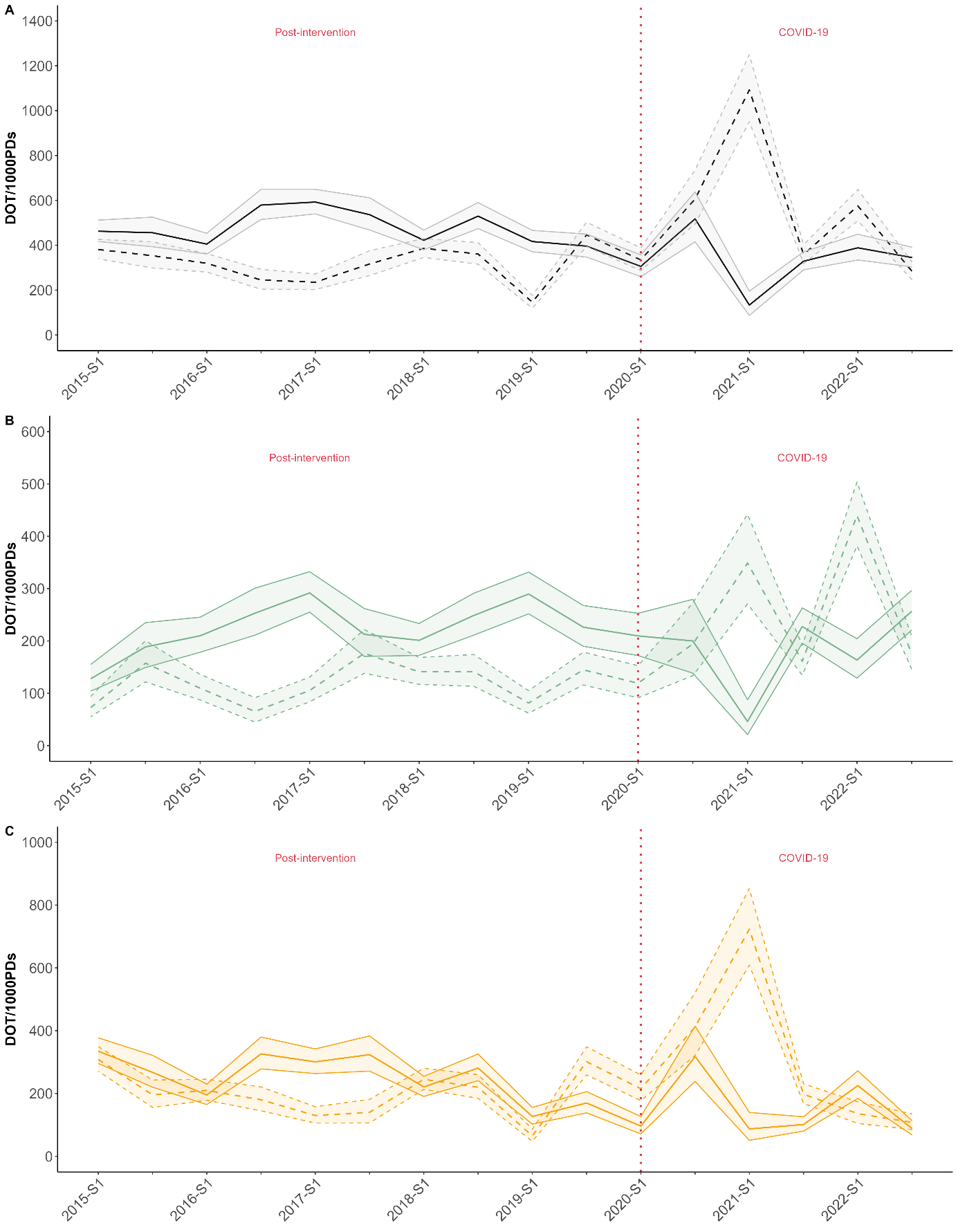
**Evolution of DOT/1000 PDs for children with lower respiratory tract infection for all the antibiotics (A) and according to the AWaRe classification: Access (B) and Watch (C) by semesters during the study period (2014-2022).

**Figure S3.**
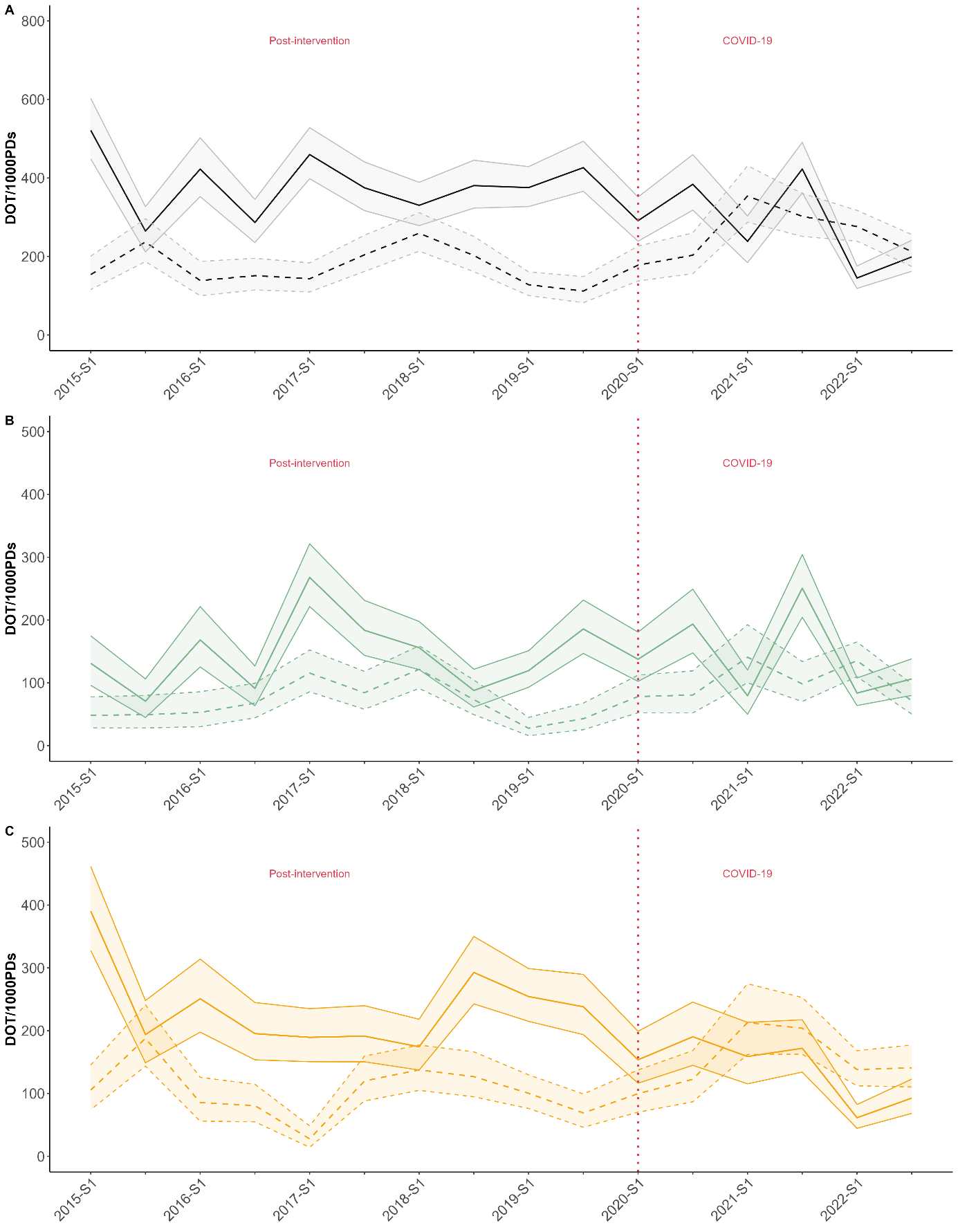
Evolution of DOT/1000 PDs for children with upper respiratory tract infection for all the antibiotics (A) and according to the AWaRe classification: Access (B) and Watch (C) by semesters during the study period (2015-2022).


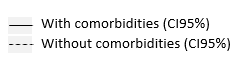

Supplement: dlae181_Supplementary_Data [file dlae181_supplementary_data.docx]
